# Multi‐modality imaging problem‐solving for pelvic splenosis on PSMA PET in a patient with recurrent prostate cancer

**DOI:** 10.1002/bco2.70090

**Published:** 2025-11-03

**Authors:** James Morgan, Anurag Anugu, Jorge D. Oldan, Michael C. Repka, Steven P. Rowe

**Affiliations:** ^1^ Department of Radiology University of North Carolina Chapel Hill North Carolina USA; ^2^ Duke University Durham North Carolina USA; ^3^ Department of Radiation Oncology University of North Carolina Chapel Hill North Carolina USA; ^4^ University of Texas Southwestern Medical Center Dallas TX USA

**Keywords:** pitfall, prostate‐specific membrane antigen, PSMA

## 1 INTRODUCTION

**FIGURE 1:**
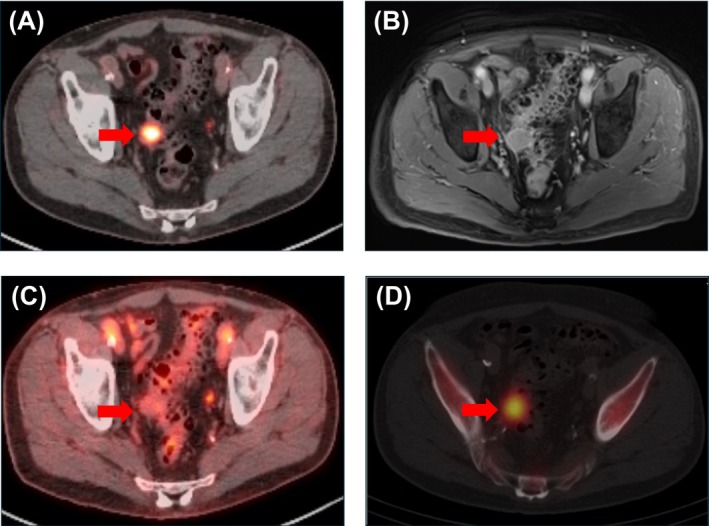
Fused PSMA PET‐CT (A), T1‐weighted fat‐saturated postcontrast MRI (B), FDG PET‐CT (C) and fused sulphur colloid SPECT‐CT (D) images of the area of pelvic splenosis, indicated by a red arrow. The lesion takes up sulphur colloid strongly (as expected for splenosis), as well as PSMA strongly, but shows moderate enhancement with gadolinium‐based MR contrast and mild uptake similar to surrounding bowel with FDG, all of which may be important in identifying these lesions in the future.

We present the case of a 73‐year‐old man with a history of remote splenectomy after trauma, Gleason 3 + 4 prostate adenocarcinoma treated with external beam radiation and 6 months of androgen deprivation therapy 11 years prior to presentation, and T3N0M0 colonic adenocarcinoma treated with hemicolectomy 5 years prior to presentation. He was found to have an increasing PSA on multiple surveillance labs up to 0.94 ng/mL prior to presentation. ^18^F‐DCFPyL prostate‐specific membrane antigen (PSMA)‐targeted positron emission tomography (PET) was performed to evaluate for recurrent disease or sites of metastasis. That scan showed mild uptake in the peripheral zone of the prostate as well as a 2.3 cm focus of moderate uptake in the sigmoid colon that was considered concerning for a malignant implant (Figure 1A). However, as splenic tissue is also known to normally express PSMA, splenosis was also included on the differential.^1^, ^2^, ^3^, ^4^

## 2 DISCUSSION

MRI was performed with a nondiagnostic evaluation of the prostate and further characterization of the colonic lesion, which appeared to arise from the distal sigmoid colon (Figure 1B). Comparison to prior imaging showed stable size over multiple prior scans, raising the possibility of a small gastrointestinal stromal tumour (GIST), an entity that is also known to express PSMA.^5^ Particularly considering the history of colon cancer, a 2‐deoxy‐2‐[^18^F]‐fluoro‐D‐glucose (FDG) PET was performed, showing a grossly stable lesion of the sigmoid colon with similar uptake to the surrounding bowel (Figure 1C). A surveillance colonoscopy was not able to identify the lesion of interest and also did not show any other evidence of colon cancer recurrence.

After discussion at the tumour board, the decision was made to perform ^99m^Tc‐sulphur colloid liver‐spleen scan with single‐photon emission computed tomography (SPECT)/CT to rule out splenosis prior to attempting tissue sampling. Although there have been cases that have indicated that heat‐damaged tagged red blood cells can have improved sensitivity for splenic tissue,^6^ that agent is not readily available at our institution, whereas ^99m^Tc‐sulphur colloid can be conveniently obtained. Ultimately, the ^99m^Tc‐sulphur colloid liver‐spleen scan showed increased uptake within the lesion, consistent with implanted splenic tissue (Figure 1D).

With widespread usage of PSMA‐targeted radiotracers, it is important to remain cognizant of the potential pitfalls that can be encountered. Similarly, a thorough review of the patient history, prior images and utilization of other imaging modalities are frequently essential to effectively troubleshoot abnormal presentations of disease and arrive at the correct diagnosis without subjecting the patient to unnecessary invasive procedures. In this case, splenosis and colonic malignancy were included on the initial differential after review of the patient history revealed the history of posttraumatic splenectomy and colon adenocarcinoma. MRI was recommended both to further characterize the region of prostatic uptake as well as the colonic mass; however, artifactual limitations of the images hindered a complete evaluation. Comparison was able to be made to prior MRI, which showed the relative stability of the colonic lesion for multiple years. That realization played a key role in steering the workup toward more indolent disease processes or anatomic variants, ultimately leading to the decision to pursue ^99m^Tc‐sulphur colloid liver‐spleen scan over invasive tissue sampling.

This case also underscores the value of maintaining familiarity with less commonly used imaging modalities such as the liver‐spleen scan when troubleshooting imaging findings. While more advanced PET radiotracers such as PSMA‐targeted agents play an increasingly central role in oncologic imaging, traditional nuclear medicine studies can offer definitive answers when initial imaging produces indeterminate findings. In cases such as this one, invasive biopsies or surgical intervention can be entirely avoided with the implementation of ‘old’ diagnostic tools.

## AUTHOR CONTRIBUTIONS

JM and SPR conceived the article. JM, AA, and SPR did primary writing on the manuscript. JDO and MCR provided critical revisions of the manuscript.

## CONFLICT OF INTEREST STATEMENT

SPR has received research funding from Progenics Pharmaceuticals, Inc., a wholly owned subsidiary of Lantheus Pharmaceuticals, Inc., the licensee of ^18^F‐DCFPyL. SPR serves as a consultant to Progenics Pharmaceuticals, Inc.
